# Influence of Initiator Concentration on the Polymerization Course of Methacrylate Bone Cement

**DOI:** 10.3390/polym14225005

**Published:** 2022-11-18

**Authors:** Grzegorz Przesławski, Katarzyna Szcześniak, Piotr Gajewski, Agnieszka Marcinkowska

**Affiliations:** Institute of Chemical Technology and Engineering, Poznan University of Technology, Berdychowo 4, 60-965 Poznan, Poland

**Keywords:** radical polymerization, kinetics, bone cements, redox-initiating system

## Abstract

*Background*: The amount of oxidant (initiator) and reductant (co-initiator) and their ratio have a significant effect on the properties of polymethacrylate bone cement, such as maximum temperature (T_max_), setting time (t_set_) and compressive strength (σ). The increase in the initiating system concentration causes an increase in the number of generated radicals and a faster polymerization rate, which shortens the setting time. The influence of the redox-initiating composition on the course of polymerization (rate of polymerization and degree of double bond conversion) and the mechanical properties of bone cement will be analyzed. *Methods*: Bone cements were synthesized by mixing a powder phase composed of two commercially available methacrylate copolymers (Evonic) and a liquid phase containing 2-hydroxyethyl methacrylate (HEMA), methyl methacrylate (MMA), and triethylene glycol dimethacrylate (D3). As an initiating system, the benzoyl peroxide (BPO) as an oxidant (initiator) in combination with a reducing agent (co-initiator), N,N-dimethylaniline (DMA), was used. Samples were prepared with various amounts of peroxide BPO (0.05%, 0.1%, 0.2%, 0.3%, 0.5% and 0.7% by weight) with a constant amount of reducing agent DMA (0.5 wt.%), and various amounts of DMA (0.25%, 0.35% and 0.5% by weight) with a constant amount of BPO (0.3 wt.%). The polymerization kinetics were studied by differential scanning calorimetry (DSC). Doughing time and compressive strength tests were carried out according to the requirements of the ISO 5833:2002 standard. *Results*: The increase in polymerization rate was due to the increase in the amount of BPO. In addition, the curing time was shortened, as well as the time needed to achieve the maximum polymerization rate. The final conversion of the double bonds in the studied compositions was in the range 74–100%, and the highest value of this parameter was obtained by the system with 0.3 wt.% of BPO. The doughing times for each BPO concentration were in the range of 90–140 s. The best mechanical properties were obtained for the cement following the initiating system concentrations: 0.3 wt.% of BPO and 0.5 wt.% of DMA. Nevertheless, all tested cements met the requirements of the ISO 5833:2002 standard. *Conclusions*: Based on the conducted polymerization kinetic studies, the best reaction conditions are provided by an initiating system containing 0.3 wt.% of BPO oxidant (initiator) and 0.5 wt.% of DMA reductant (co-initiator). A decrease in the DMA amount caused a decrease in the polymerization rate and the amount of heat released during the reaction. The change in BPO and DMA concentrations in the composition had little effect on the doughing time of the studied bone cement. The cements showed similar doughing times, ranging from 90–225 s, which is comparable to the bone cement available on the market.

## 1. Introduction

Extending the life of the population, aging societies, as well as changing the lifestyle, diet and limiting physical activity resulting from technological development, mean that more and more people suffer from complex bone fractures resulting from traumatic events, such as sports injuries, car accidents, falls, or various diseases, such as bone cancer, bone degeneration and osteoporosis [1,2].

In each of these cases, adequate internal or external stabilization of the damaged parts is required. Internal stabilization of damaged areas can be performed with, among others, wires, rods, screws, plates or bone cement. Bone adhesives are biomaterials designed to stabilize complex fractures as well as to fix implants [3], and they can be classified as structural adhesives, which are distinguished by high strength and resistance to various types of loads. There are static loads, such as: shear, compression, tension, torsion or dynamic loads, such as impact strength [4]. Basically, the bone cement should be able to form strong connections with the bone tissue and be responsible for proper stabilization. Among them, we can distinguish various types of materials (both synthetic and natural), but none of them meet all the requirements for this type of material.

The cements available on the market are acrylate cements (mainly based on poly(methyl methacrylate), PMMA), cured in the patient’s body by polymerization, which is characterized by, among others, good mechanical strength, bio-compatibility and quick rehabilitation of patients after surgery [5]. Unfortunately, these types of materials also have a number of disadvantages, such as moderate adhesion, temperature rise during hardening, high content of residual monomer [3,6,7], polymerization shrinkage, poor penetration of the bone structure, no bio-degradability, poor antibacterial properties, and weak or no contrasting properties in imaging [8].

The polymerization of methacrylates is a highly exothermic reaction. The heat released during this process may cause the system temperature to rise to 48–105 °C at the interface of the bone/cement [9] and to 80–124 °C in the bone cement. This, in many cases, may cause necrosis of the bone tissue, where the threshold level for thermal damage is estimated to be 48–60 °C and depends on the time of the exposure [10,11]. This damage may cause implant loosening [12,13]. Moreover, the unreacted monomer remaining after polymerization may be toxic and cause necrosis of the soft tissues surrounding the surgical site [11,14,15]. Due to these reasons, the properties and performance of acrylic bone cement depend on the kinetics of polymerization.

There are many reports on the polymerization kinetics of acrylic bone cements [10,11,13,16,17,18,19,20,21,22]. In practice, bone cements are applied into a patient’s body under isothermal conditions, thus, the study of polymerization under isothermal conditions by differential scanning calorimetry (DSC) will be a more practical method than a dynamic scan experiment.

The amount and ratio of oxidant and reductant have a significant effect on the properties of bone cement, such as the maximum temperature (T_max_) determined during the curing of bone cement, setting time (t_set_) and compressive strength (σ). Two of these parameters, setting time and compressive strength, are inversely related to each other, and obtaining optimal t_set,_ causes a decrease in compressive strength [23,24,25]. The increase in the initial system concentration causes an increase in the number of generated radicals and a faster polymerization rate, which shortens the setting time. Moreover, more single polymer chains will be obtained during the polymerization, and the average molecular weight of the polymer will decrease, which will affect the mechanical properties.

Nussbaum and co-workers in their work [26] showed that the increase in the amount of benzoyl peroxide (BPO) from 0.5 to 2.75 wt.% at a constant concentration of N,N-dimethyl-p-toluidine (DMPT) (0.2 wt.%) caused the setting time to be shortened from 28.30 to 9.26 min. Likewise, increasing the amount of DMPT from 0.2 to 4.9 wt.% at a constant BPO content (0.5 wt.%) shortened the setting time from 28.20 to 5.50 min, but no significant changes were observed at the maximum curing temperature. Moreover, the increase in the concentration of BPO and DMPT resulted in an increase in the compressive strength. Cements with the lowest concentrations of BPO and DMPT were significantly weaker than other cement.

The influence of the amount of the oxidant and the reductant on the parameters of bone cement was investigated by Madigan and coworkers [27]. In their work, they increased the amount of co-initiator (DMPT) from 0.8 to 2.4% *v*/*v* and the amount of initiator (BPO) by increasing the amount of commercial PMMA-styrene co-polymer powder, which also contained BPO in the manufacture’s amounts. The results show that an increase in the amount of DMPT with a constant amount of BPO affects the setting time but does not have a significant effect on the maximum temperature of the curing process or compressive strength. Meanwhile, an increase in the amount of co-polymer with BPO with a constant amount of DMPT caused an increase in compressive strength and a decrease in maximum temperature.

Yang [20] studied the effect of 2-hydroxyethyl methacrylate (HEMA) and ethylene glycol dimethacrylate (EGDMA) on the polymerization process of commercial bone cement Simplex^®^-P in isothermal conditions as well as the addition of tricalcium phosphate (TCP) on polymerization kinetics [22]. The addition of HEMA caused a longer induction time with an increasing HEMA content and a sharp increase in heat release. Moreover, despite the longer time for polymerization, the conversion of the monomer fraction was obtained at the same level (100%). In the second step, we added the crosslinking monomer EGDMA in the amount of 0.3% to the commercial bone cement modified by 15 wt.% of HEMA. Kinetic studies of these compositions showed that with an increasing EGDMA content, the initial time of polymerization decreases, but the heat of polymerization increases. Moreover, an increase in the initial time and heat release with increasing TCP concentration was observed.

Borzacchiello et al. [10] studied the polymerization kinetics of poly(ethylmethacrylate)-based bone cement with hydroxyapatite (HA) and n-butylmethacrylate (nBMA) monomers in isothermal conditions at 20 °C. They showed that the temperature at the bone/cement interface was lower than 50 °C and the conversion was over 90%.

The polymerization in isothermal conditions at 37 °C of commercial bone cements Sulfix-6 and Zimmer LVC 60/30 was studied by Migliaresi and co-workers [11]. The maximum polymerization temperature of the system was obtained approximately 6.6 or 5.1 min after mixing, and 99% of the conversion was achieved for both types of cements within 10 min.

Methacrylates are chemical compounds from the group of unsaturated esters having in their structure a double bond between two carbon atoms, which is characterized by high reactivity. This makes these compounds easy to polymerize. The polymerization of multi-functional monomers (with two or more methacrylate groups in one molecule) provides an easy way to produce highly crosslinked polymer networks for a variety of applications. The high crosslinking density of these systems increases the mechanical and thermal stability of the polymer [28,29]. On the other hand, the polymerization of monomethacrylates (with one methacrylate group in the molecule) results in a linear polymer structure.

In the case of homopolymerization of ethylene glycol dimethacrylate, the reaction characteristics and structure of the polymers obtained are radically different from those obtained in copolymerization with monomethacrylates. During the copolymerization of ethylene glycol monomethacrylates with small amounts of dimethacrylate crosslinking monomer, homogeneous hydrophilic materials are obtained, while homopolymerization of dimethacrylates usually yields heterogeneously glassy polymers [30].

Furthermore, in contrast to reactions leading to weakly crosslinked polymers, the homopolymerization of dimethacrylates often shows a maximum conversion lower than 100%. Homopolymerization of dimethacrylates shows a monotonic increase in radical concentration. Experimental studies of the free radical crosslinking of methacrylates have shown that the reaction characteristics and network structure depend on the concentration of the crosslinking monomer [30].

Multi-functional systems exhibit complex features, i.e., auto-acceleration and auto-deceleration, that limit double bond conversion; the chain termination reaction is controlled by a diffusion reaction mechanism from the beginning of polymerization [28]. The polymerization kinetics are complex due to the change in the mobility of the medium as the reaction progresses. Propagation and termination reactions are chemically controlled by low degrees of double bond conversion. However, network formation restricts segmental movements of radicals, and termination is limited only by the diffusion reaction mechanisms. A decrease in the termination rate leads to an increase in the number of growing radicals (macro-radicals) and a corresponding increase in the propagation rate. The described increase in polymerization rate has been termed auto-acceleration and is often found in dimethacrylates polymerization. As the reaction proceeds, the environment becomes even more confined—due to the increase in viscosity and density of the forming polymer network, the propagation reaction also becomes diffusion controlled. The rate of polymerization drops significantly as both propagation and termination are limited by diffusion reactions. This decrease in the polymerization rate is called the auto-deceleration effect. Diffusion-controlled polymerization kinetics lead to conversions less than unity, which occur especially in glassy systems [28,29,31,32].

This work will investigate the influence of redox-initiating composition on the course of the polymerization (rate of polymerization and degree of double bond conversion) of the methacrylate bone cement consisting of a powder and a liquid phase. Additionally, the mechanical properties (compressive strength) of prepared bone cements samples will be analyzed. As mentioned above, the synthesized materials should meet the requirements of bone cements, so they should be characterized by a low-temperature rise during hardening, a very low concentration of unreacted monomers, and a high mechanical strength.

## 2. Materials and Methods

### 2.1. Materials

The powder phase was composed of two commercially available methacrylate co-polymers (Evonic, Essen, Germany) in the ratio 80:20. The liquid phase contained 70 wt.% 2-hydroxyethyl methacrylate (HEMA), 15 wt.% methyl methacrylate (MMA) and 15 wt.% triethylene glycol dimethacrylate (D3) (Sigma Aldrich, St. Louis, MO, USA). Benzoyl peroxide (BPO) was used as an oxidant (initiator) in combination with a reducing agent (co-initiator), N,N-dimethylaniline (DMA) (Sigma Aldrich, St. Louis, MO, USA).

### 2.2. Sample Preparation

The liquid phase was prepared by incorporating DMA in the amounts of 0.25, 0.35 or 0.50 wt.% (2.292, 3.209 and 4.584 µmol, respectively) to earlier prepared monomers mixture (HEMA+MMA+D3). The concentration of DMA was calculated in relation to the mass of the liquid phase of cement composition, i.e., mass of monomers. The mixtures were homogenized on a mechanical shaker MS 3 digital (IKA, Staufen im Breisgau, Germany) to obtain homogenous compositions. The powder phase was prepared separately by mixing co-polymers and then by incorporating of the peroxide BPO (initiator) in several amounts (0.05 wt.% = 0.229 µmol, 0.1 wt.% = 0.459 µmol, 0.2 wt.% = 0.917 µmol, 0.3 wt.% = 1.376 µmol, 0.5 wt.% = 2.293 µmol and 0.7 wt.% = 3.211 µmol) according to the mass of the liquid phase. The powder phase was mixed with the liquid phase in a mass ratio 1:1.25.

### 2.3. Polymerization Kinetics

The prepared powder phase was mixed with the liquid phase, and then the mixture was inserted into a DSC sample pan in the amount of 30 ± 1 mg. Next, the crucible was placed in the chamber of the differential scanning calorimeter (DSC) apparatus (along with an empty crucible as a reference) and washed with an inert gas (Ar) at a flow rate of 20 mL/min. Two minutes elapsed for each sample from the start of mixing to placing the crucible with the sample in the DSC chamber. The inert gas was passed through the chamber for one minute before and during the measurement. Thus, the DSC measurements were started every time after three minutes from the beginning of mixing the liquid phase with the powder phase. The test was carried out using the DSC 6000 instrument (Perkin–Elmer, Waltham, MA, USA) under isothermal conditions (25 °C). The reaction exotherm, in normalized values, W/g, was recorded as a function of the reaction time until a constant value of the measured parameter was obtained. The reaction rate (*R_p_ = dx*/*dt*) was then calculated from the rate of the heat release (*d(*Δ*H)/dt*) from Equation (1).

(1) $$R_{p}=\frac{1}{\Delta H_{T}}\frac{d\left(\Delta H\right)}{dt}\;\;$$

where Δ*H_T_* denotes the total reaction enthalpy. The degree of double bond conversion was calculated by integrating the area between the DSC thermograms and the baseline. The baseline was obtained for the measurement performed after complete polymerization, when no change in the temperature was observed during the reaction. For the calculation of the polymerization rate and degree of double bond conversion, the heat of polymerization of one methacrylate group, 56 kJ/mol, was used [33]. In order to estimate the total amount of heat released during the reaction, the weight of the samples was corrected by the weight of the co-polymers mixture, which does not participate in the polymerization reaction.

### 2.4. Doughing Time

According to ISO 5833:2002, the standard [34] doughing time is the time when the gloved finger first separates cleanly from the cement. The powder and liquid phases were mixed in a ratio of 1:1.25, and after 1 min, the surface of the mixture was gently touched with an un-powdered, non-water-rinsed gloved finger. It was observed whether fibers formed between the cement and the glove as the finger left the surface. The probing process was repeated at maximum intervals of 15 s to the doughing time. The procedure was conducted for two samples (according to the ISO 5833:2002 standard [34]). If the two doughing times differed by more than 30 s, the probing process was repeated for the remaining two units of cement.

### 2.5. Compressive Strength

The prepared mixture of powder and liquid phases of bone cement was placed in cylindrical plastic (PE) molds with dimensions: 12 mm high and 6 mm in diameter. Polymerization was carried out in a laboratory dryer (Memmert SF75, Memmert GmbH + Co. KG, Schwabach, Germany) for 1 h at 36.6 °C and constant humidity (30%). After that, the resulting bone cement was taken out of the mold.

Compressive strength (σ) testing was performed in accordance with the requirements of ISO 5833:2002 [34]. Additionally, 24 ± 2 h after mixing the two phases (powder and liquid) of the cement, the obtained samples were subjected to compressive strength testing on a Zwick/Roell Z020 testing machine (Zwick AG, Ulm, Germany). A constant crosshead speed of 22.5 mm/min was used. The measurement was carried out until the specimen broke and was repeated at least five times. The obtained results of compressive strength and Young’s modulus were recalculated using a computer program dedicated to the Zwick/Roell Z020 testing machine.

## 3. Results and Discussion

### 3.1. Polymerization Kinetics

**Scheme 1:**
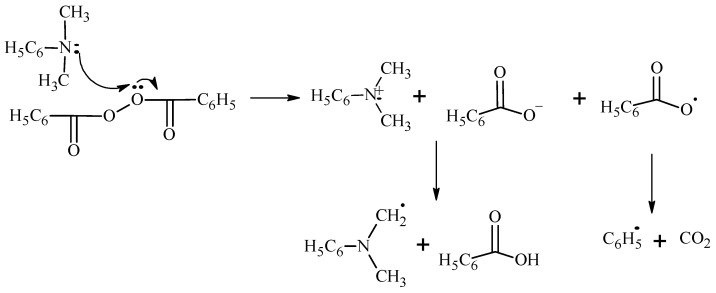
Mechanism of benzoyl peroxide decomposition in the presence of N,N-dimethylaniline (redox initiation).

In this work, two different methacrylate co-polymers were used as the powder phase and the mixture of monomethacrylate and dimethacrylate monomers as the liquid phase to prepare bone cement that meets the requirements of the ISO 5833:2002 standard [34]. The new system composition was used, so it is important to know how the initiating system will influence its polymerization process. As part of initiating the system of benzoyl peroxide with N,N-dimethylaniline, a reducing agent (co-initiator, DMA) was used. The mechanism of the decomposition of peroxide BPO in the presence of tertiary amine DMA with the formation of initiating radicals is presented in Scheme 1. This is a complex reaction sequence, and it cannot be described with any Arrhenius law. The redox initiating system is often used for the initiation of the polymerization reaction during the curing process of the bone cement. The amount of the initiating system determines the rate of the polymerization and conversion of the reactive groups of the monomers, and thus, the temperature of the curing process. The temperature of the polymerization process (the exothermic reaction) is crucial when the cement is cured in the patient’s body. This temperature should not exceed 90 °C as it may cause tissue necrosis. Moreover, the duration time of this high temperature should be as short as possible. At the same time, the obtained cement should be mechanically strong enough to fulfill its task. Thus, the influence of the benzoyl peroxide as well as the reducing agent DMA concentration on polymerization kinetics of bone cement curing was investigated as well as on the compressive strength of the obtained material. During the tests, a low concentration of DMA than BPO was used to avoid a situation where unreacted amine would remain in the cement after polymerization, as it may have a toxic effect on the human body. Aromatic amines have a wide range of toxic effects, but as the literature shows, the DMA toxicity is reversible. The cells do not die but slow down their replication cycle, which does not affect the bone tissue repair process [35]. Thus, these compounds are used in bone cements. In addition, the peroxide BPO and reducing agent DMA react (Scheme 1) [36] in a molar ratio of 1:1, and in this study, the BPO:DMA ratios were chosen to provide almost a full conversion of both BPO and DMA.

The polymerization reaction consists of three steps: initiation, propagation and termination. Each of these steps influences the whole polymerization process. As is known, the concentration of the initiating system (initiator and co-initiator, oxidant-reductant) in redox polymerization influences the rate of the initiation reaction (Equation (2)), and thus, influences the polymerization process (Equation (3)) [37]:

(2) $$R_{i}=k_{d}\left[reductant\right]\left[oxidant\right]$$

(3) $$R_{p}=k_{p}\left[M\right]{\left(\frac{k_{d\;}\left[reductant\right]\left[oxidant\right]}{2k_{t}}\right)}^{0.5}\;$$

In many polymerization processes initiated by the redox system, a monomer may be involved in the initiation process. It can be found in the literature that N,N-dimethylaniline or its derivatives, such as, for example, N,N-dimethyl-p-toluidyne, initiate the polymerization of methyl methacrylate by charge-transfer complex formation, alone without benzoyl peroxide [38,39]. This is due to the charge transfer complex that forms between the amine and the monomer. In this case, the rate of the polymerization process is consistent with Equation (4):

(4) $$R_{p}=k{\left[M\right]}^{1.5}{\left[amine\right]}^{0.5}\;$$

In this work, the UV spectroscopy measurements (not shown) of the mixtures of methyl methacrylate as well as monomer compositions with DMA were performed, and for comparison, the spectrum of the mixture of DMA with ethanol was also measured. In all spectra, the absorbance peak was observed at 300 nm, and no shift in the absorption peak was observed in the mixtures of monomers with DMA compared to DMA—ethanol mixtures. Thus, there is no charge transfer complex formation in the investigated systems. Moreover, measurements of the course of polymerization of bone cement compositions (powder with liquid phases) with DMA without BPO show no heat release (DSC measurements, not shown). Therefore, the initiation of polymerization by DMA itself does not take place. Therefore, in the investigated systems, the rate of polymerization is described by Equation (3).

**Figure 1:**
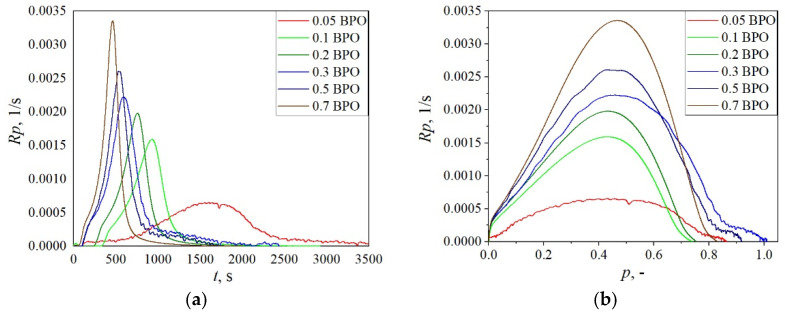
The kinetic curves of the polymerization of bone cement compositions containing various amounts of peroxide BPO and 0.5 wt.% of DMA reductant as a function of polymerization rate (*R_p_*) from (**a**) reaction time (*t*) and (**b**) double bond conversion (*p*).

In the first stage of work, the influence of the amount of the benzoyl peroxide on the polymerization kinetics was analyzed. The concentration of the amine (DMA) was constant. In the second stage, the influence of the amount of the reducing agent DMA on the polymerization process was examined. Figure 1 shows the kinetic curves obtained for compositions with different concentrations of the peroxide BPO in the range 0.05–0.7 wt.% and a constant concentration of DMA reductant (0.5 wt.%).

With the increase in the amount of BPO, the heat release during the polymerization process increased. However, the time needed for the curing process was reduced, as was the time needed to reach the maximum rate of polymerization (*t^Rm^*) (Figure 1a). The maximum rate of the polymerization reaction (*R_p_^max^*) was obtained in the shortest time *t^Rm^* for a composition with 0.7 wt.% of BPO (*R_p_^max^* = 0.00336 1/s), and the time of the maximum rate of the reaction was *t^Rm^* = 462 s (7.7 min). The lowest *R_p_^max^* value of 0.000546 1/s was obtained for a composition with 0.025 wt.% of BPO. This polymerization rate was achieved at time *t^R^*^m^ equal to 2028 s (33.8 min).

**Figure 2:**
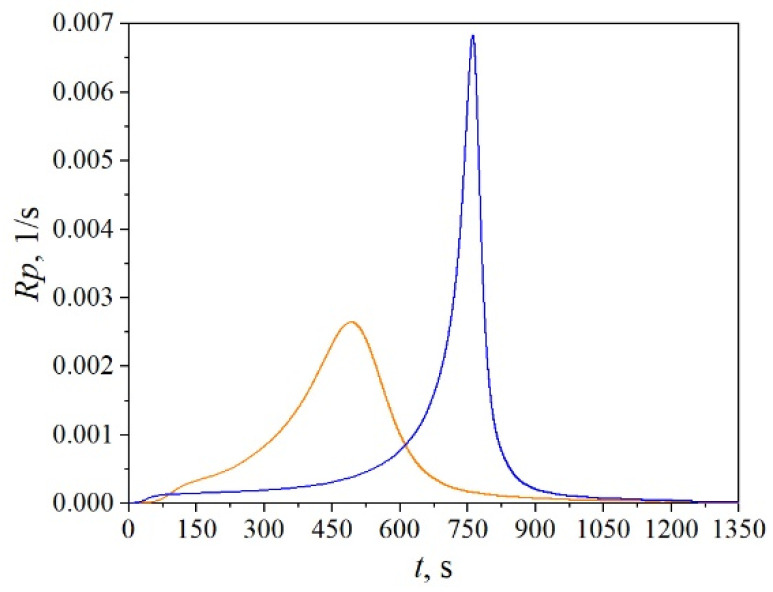
Kinetic curves of polymerization of bone cement composition (orange line) and monomers mixture (blue line) containing 0.1 wt.% of BPO initiator and 0.5 wt.% of DMA co-initiator as a function of polymerization rate (*R_p_*) from reaction time (*t*).

For all studied compositions, the polymerization process is characterized by the inhibition period in which no reaction takes place. This can be caused by reaction inhibition exerted by oxygen as well as inhibitors. Oxygen was introduced into the samples during their preparation by powder and liquid phase mixing. The inhibitor was not removed from the monomers before sample preparation; they were used as received. Thus, the inhibitor used for monomer stabilization can be the cause of the inhibition period occurrence. The inhibition time allowed, however, for performing measurements under stable, repeatable conditions. This period decreases with increasing concentrations of benzoyl peroxide. The stationary state of the polymerization reaction is observed and is the longest for the composition containing the smallest amount of the BPO (0.05 wt.%) and for the rest of the studied samples (compositions containing ≥0.1 wt.% of peroxide BPO), which are visible as inflections on the kinetic curves. The high viscosity of the compositions (they contain a high amount of the co-polymer mixture) as well as the formation of the polymer network during the reaction (due to the presence of dimethacrylate monomer in the composition) cause the limitation of radicals diffusion, thus, auto-acceleration of the polymerization starts almost from the beginning of the reaction. When only a mixture of monomers, without the addition of co-polymers, was polymerized, the auto-acceleration of the polymerization reaction began after a longer reaction time. This is caused by the lower viscosity of the curable mixture than in the case of a mixture of monomers and co-polymers (Figure 2). Moreover, the exothermic peak is lower for polymerization with co-polymers in the composition, which indicates that the heat release (increase in temperature) during the curing process is lower, which may reduce the likelihood of surrounding tissue necrosis. After a stationary state rapid increase in the polymerization rate, the greater the concentration of BPO in the composition is observed. This indicates that the temperature of the cement during curing may rise to higher values for compositions with higher amounts of the initiator. Higher initiator concentration in the system contributes to a higher propagating radical concentration, which, therefore, leads to shorter chain lengths and a higher concentration of microgels formed, which can influence heterogeneity in the cement structure. This, in turn, may affect the mechanical resistance of the materials obtained.

The final double bond conversion (*p^f^*) of the studied compositions is within the range of 74–100%, thus, the highest value of this parameter is obtained by the system with 0.3 wt.% of BPO and the lowest with 0.1 wt.% of the initiator. The obtained relations show that the *p^f^* depends not on the polymerization rate or the reaction time, but on the BPO:DMA ratio. Based on these results, we can select a mixture containing 0.3 wt.% of BPO as the one containing the optimal amount of the initiator. Since 0.5 wt.% of the co-initiator was used, the molar ratio of the initiating system was approximately 1:4 (BPO:DMA), so there were four co-initiator molecules per one initiator molecule.

**Figure 3:**
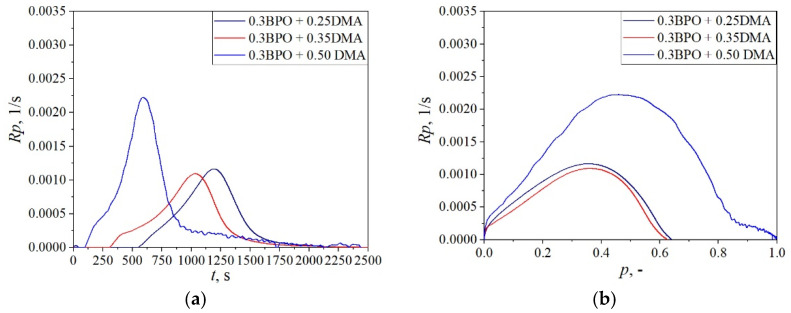
Kinetic curves of polymerization of bone cement compositions containing various amounts of DMA co-initiators and 0.3 wt.% of BPO initiators as a function of polymerization rate (*R_p_*) from (**a**) reaction time (*t*), and (**b**) double bond conversion (*p*).

Thus, in the second stage of the kinetics studies, this optimal concentration of the benzoyl peroxide and different amounts of the co-initiator N,N-dimethylaniniline were used to find the best ratio of the reductant:oxidant for the polymerization process of the studied bone cement composition (Figure 3.).

The concentration of BPO was set as 0.3 wt.%, and for DMA, it was set as 0.25 wt.%, 0.35 wt.% and 0.5 wt.% of monomer mass, which corresponds to approximately 1.7, 2.3 and 3.3 co-initiator molecules per one initiator molecule, respectively.

The heat release during polymerization increased with increasing amounts of DMA, as can be seen from the kinetic curves presented in Figure 2. The time of the reaction decreased. Thus, the dependencies are similar to those obtained for different concentrations of BPO initiator in the system. The higher the concentration of the initiator or co-initiator, the higher the polymerization rate and the shorter the time of the process. The maximum rate of the polymerization reaction (*R_p_^max^*) was obtained in the shortest time for a composition containing 0.5 wt.% DMA (*R_p_^max^* = 0.00198 1/s), and the time of the maximum rate of reaction (*t^Rm^*) was 580 s (9 min 40 s). The other two compositions have similar maximum polymerization rate values, but the time needed to achieve this is longer for a composition containing 0.25 wt.% of DMA by approx. 3 min (*t^Rm^* = 1173 s).

The inhibition period is longer when a lower concentration of DMA is used for the polymerization process, as was the case with various amounts of the BPO initiators.

The highest final conversion (*p^f^*) was achieved by the composition containing 0.5 wt.% of the co-initiator, and the smaller amount of DMA causes a decrease in this parameter by almost 40%. Thus, the concentration of DMA had a greater impact on the degree of double bond conversion of the studied composition than BPO.

Based on the conducted polymerization kinetic studies, it can be concluded that the best reaction conditions are provided by a system containing 0.3 wt.% of BPO initiator and 0.5 wt.% of DMA co-initiator. However, bone cement must also have the appropriate production parameters (doughing time) to be able to properly perform the patient’s operation and mechanical strength to ensure a sufficiently strong bone-bone fixation or bone implant. Therefore, the following sections present the results of the measurements of the doughing time and compressive strength of the prepared bone cements.

### 3.2. Doughing Time

**Figure 4:**
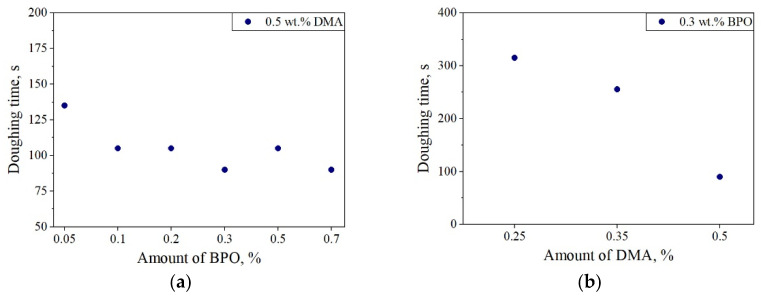
Doughing time of bone cement containing (**a**) 0.5 wt.% of DMA and different amounts of BPO initiators, and (**b**) 0.3 wt.% of BPO and various concentration of DMA co-initiator.

The doughing time is a very important parameter that describes how much time it will take before the bone cement starts hardening, and it is described as the time that passes from mixing the two phases of bone cement to applying them inside the patient body. The doughing times obtained for compositions containing different amounts of a BPO initiator (in the range 0.05–0.7 wt.%) and constant amounts of DMA co-initiator (0.5 wt.%) are presented in Figure 4a, and a DMA co-initiator containing different DMA concentrations (in the range 0.25–0.5 wt.%) with constant initiator content (0.3 wt.%) are presented in Figure 4b.

The doughing time for each BPO concentration was in the range of 90–140 s. Additionally, it appears that there is a slight decrease in this parameter with the rising concentration of BPO for compositions containing 0.05–0.2 wt.% of this compound, and then for higher concentrations of BPO, no dependence of doughing time on the concentration is observed. This range is comparable to the commercially available bone cement with a doughing time of 75–210 s. These results are consistent with the measurements of the course of the polymerization reaction using the DSC method, where, with increasing BPO concentration in the composition, a reduction in the time needed to obtain the maximum polymerization rate was observed.

Then, the concentration of DMA was changed from 0.25 wt.% to 0.5 wt.% of monomers mass, with a constant amount of BPO (0.3 wt.%). Increasing concentration of DMA in composition caused substantial decrease in doughing time from 325 s for 0.25 wt.% of DMA to 90 s for composition with 0.5 wt.% of DMA. Thus, if a longer doughing time is needed, a lower amount of DMA co-initiator can be used. However, this will significantly affect the polymerization kinetics, as presented in the previous section.

### 3.3. Compressive Strength

**Figure 5:**
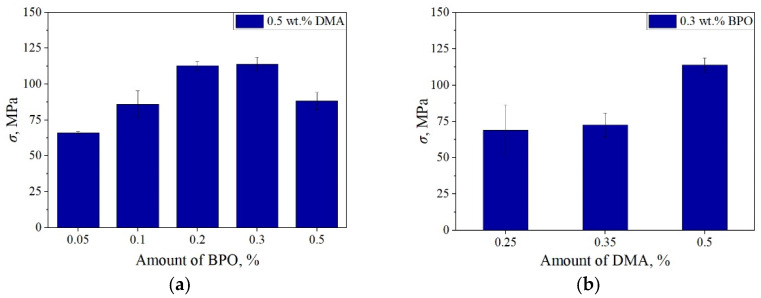
Maximum compressive strength of bone cement with (**a**) 0.5 wt.% of DMA and different amounts of the BPO initiator, and (**b**) 0.3 wt.% of BPO and various concentrations of the DMA co-initiator.

The mechanical properties, i.e., compressive strength, of prepared cement with various concentrations of initiator or co-initiator were studied. The maximum value of compressive strength as a function of initiator BPO concentration is presented in Figure 5a, and as a function of co-initiator DMA concentration is presented in Figure 5b.

The initial increase in BPO content in the studied system, within the range of 0.05–0.3 wt.%, causes an increase in the mechanical strength (maximum compressive strength) of the obtained bone cements, while in the case of using a BPO concentration of 0.2 and 0.3 wt.% in the mixture, materials with the same compressive strength are obtained. A further increase in initiator concentration (0.5 wt.% of BPO) caused a decrease in the discussed parameter, what can be associated with the heterogeneity of the formed polymer network. In the case of testing the effect of amine concentration (DMA) on the compressive strength of bone cement, the material with the best properties was obtained with a co-initiator content of 0.5 wt.%. Therefore, also in the case of mechanical properties, the cement with the best properties was obtained when the initiating system was used with the following concentration: 0.3 wt.% of BPO and 0.5 wt.% of DMA. In this case, the doughing time as well as the time needed to reach the maximum rate of polymerization are optimal, the degree of double bond conversion is close to 1, and the compressive strength is within the ISO 5833:2002 standard [34]. The obtained results are promising, however, further studies of the properties of the obtained materials are required.

## 4. Conclusions

In this study, the effect of changing the amount of the initiator and the initiator’s ratio on the polymerization of methacrylic bone cement was investigated by the DSC method under isothermal conditions. The increasing amount of BPO at a constant amount of DMA (0.5 wt.%) caused a significant increase in the polymerization rate and in the amount of heat released. In the case of double bond conversion, the value of this parameter was influenced by the BPO:DMA ratio, and the highest degree of conversion was obtained for a system with a BPO:DMA weight ratio of 0.3:0.5. The degree of double bond conversion should be as high as possible (preferably 100%) due to the toxic effect of the monomers released from the cement in the patient’s body.

A decrease in the DMA amount caused a decrease in the polymerization rate and the amount of heat released during the reaction. Which is the desired effect due to the possibility of tissue damage (necrosis) caused by the high curing temperature of the cement (due to an exothermic reaction). However, on the other hand, the increasing amount of DMA increased the conversion of double bonds, which is also very important from the point of view of cement hardening in the human body. Therefore, it is necessary to find the optimal amount of initiator and co-initiator, which will allow for obtaining a material with the best curing properties, on the one hand with a low heat of hardening, and on the other hand, with a high degree of conversion. In this case, the best-performing cement was obtained with 0.3 wt.% of BPO and 0.5 wt.% of DMA.

The change in BPO and DMA concentrations in the composition has an insignificant impact on the doughing time of bone cement. The cement with the highest amount of BPO in the composition, i.e., 0.7 wt.%, is characterized by the shortest doughing time. However, the doughing times of the tested bone cements are similar and range from 90 to 225 s, which is comparable to commercial bone cement. Similarly, in the case of DMA, an increase in doughing time was observed with a decreasing amount of DMA. Reducing the amount of the DMA activator in the redox initiating system can reduce the amount of radicals generated during the polymerization process and slow down the polymerization reaction.

## Data Availability

The data presented in this study are available upon request from the corresponding author.
